# Long Life

**Published:** 1884-11

**Authors:** 


### LONG LIFE.

THE honors paid to aged people are evidence that mankind, as a rule, long for many years. To speak contemptously or even indifferently of life, is, for us, a sign of sentimental affectation. Medical science is encouraged to do its utmost to prolong life to the last moment. We measure the gain of man under civilization by the increased average duration of existence in large populations. In all communities, in all communions, men are planning and trying to live to the full term of their days. Under all circumstances, in all conditions, existence is tacitly assumed to be a boon. The old man—infirm, subject to painful attacks of sickness, alone, or the same as alone in the word, the wife of his bosom dead, childless, too—clings to life. He is no doubter of immortality; he accepts the church tradition on that point without misgiving. But this life he has become attached to in the course of many years. Its comforts are solid; its satisfactions are tangible; he is wonted to the dear old ways; he likes the dear old people; here he will stay as long as he can, and will not welcome death as a dellverance. The life beyond may be very good after its kind, but it is not the kind with which he is familiar. The desire for long life is not inconsistent with belief in a happy immortality. Spiritualists, whose persuasion of the reality of a future state of existence is as clear and firm as their faith in the present, are yet found clinging to the present as if it were of prime value. And it is natural that they should. The world is a good thing, better than any of us knows. He never lived who appreciated it at its worth. He never lived who used the smallest fraction of its resources. The artist almost despairs of depicting the beauty of its most familiar objects; the man of science exhausts himself in efforts to teach the secret of its perfect order; the philosopher invents system after system to explain the reason of its phenomena; philosophy itself is but an attempt to get at the cause of its every day effects.

Life is a good thing. Length of years ought to be wished for as a privilege. The world is crowded with satisfactions that only the sheerest folly will despise. Those only, who have never known what life is worth, speak of it as worth nothing. There is nothing better than this world, considered apart from the folly and sin that abuse and corrupt it. There is no greater privilege than long life in it, within the conditions that secure power of appreciation and reasonable enjoyment. The man who lives seventy or eighty years, with fair strength, wholesome appetite, even moderate capacity for enjoyment, a faculty for appreciating whatever is most worth having, and means sufficient to give him command of what he can enjoy, is in a high sense an enviable man. Such a one, the ancients would have said, the gods love.

**The Parasite of Malaria.**—The observations of M. Richard seem to affirm those of Leverau; he found in the red corpuscles of the blood of persons suffering from acute malaria a parasite of oscillating form moving very rapidly, and sometimes disengaging itself from the globule. These parasites have been met with in a number sufficiently large to obstruct the capillary vessels, and to explain many of the symptoms of intermittent fevers. It has also been proved that the culture of these parasites in a fertile gelatine basis can be brought to an immediate cessation if a two per cent quinine solntiou is added.

